# A phase I study of pexidartinib, a colony-stimulating factor 1 receptor inhibitor, in Asian patients with advanced solid tumors

**DOI:** 10.1007/s10637-019-00745-z

**Published:** 2019-03-02

**Authors:** Jih-Hsiang Lee, Tom Wei-Wu Chen, Chih-Hung Hsu, Yu-Hsin Yen, James Chih-Hsin Yang, Ann-Lii Cheng, Shun-ichi Sasaki, LiYin (Lillian) Chiu, Masahiro Sugihara, Tomoko Ishizuka, Toshihiro Oguma, Naoyuki Tajima, Chia-Chi Lin

**Affiliations:** 1 National Taiwan University Hospital, Hsin-Chu Branch No. 25, Lane 442, Sec. 1, Jingguo Rd, Hsinchu City, 300 Taiwan; 2 National Taiwan University Hospital, 7 Chung Shan S Rd, Taipei, 10002 Taiwan; 3 Graduate Institute of Oncology, National Taiwan University College of Medicine, 7 Chung Shan S Rd, Taipei, 10002 Taiwan; 4 Daiichi Sankyo Co., Ltd., 1-2-58 Hiromachi, Shinagawa-ku, Tokyo, 140-8710 Japan; 5 Daiichi Sankyo Co., Ltd., 7F-1, No. 308, Sec. 2, Bade Rd, Zhongshan Dist., Taipei City, 104 Taiwan; 6 Graduate Institute of Clinical Medicine, National Taiwan University College of Medicine, 7 Chung Shan S Rd, Taipei, 10002 Taiwan

**Keywords:** Tenosynovial giant cell tumor, Pexidartinib, Pharmacokinetics, Safety, Solid tumors

## Abstract

*Background* Pexidartinib, a novel, orally administered small-molecule tyrosine kinase inhibitor, has strong selectivity against colony-stimulating factor 1 receptor. This phase I, nonrandomized, open-label multiple-dose study evaluated pexidartinib safety and efficacy in Asian patients with symptomatic, advanced solid tumors. *Materials and Methods* Patients received pexidartinib: cohort 1, 600 mg/d; cohort 2, 1000 mg/d for 2 weeks, then 800 mg/d. Primary objectives assessed pexidartinib safety and tolerability, and determined the recommended phase 2 dose; secondary objectives evaluated efficacy and pharmacokinetic profile. *Results* All 11 patients (6 males, 5 females; median age 64, range 23–82; cohort 1 *n* = 3; cohort 2 *n* = 8) experienced at least one treatment-emergent adverse event; 5 experienced at least one grade ≥ 3 adverse event, most commonly (18%) for each of the following: increased aspartate aminotransferase, blood alkaline phosphatase, gamma-glutamyl transferase, and anemia. Recommended phase 2 dose was 1000 mg/d for 2 weeks and 800 mg/d thereafter. Pexidartinib exposure, area under the plasma concentration-time curve from zero to 8 h (AUC_0-8h_), and maximum observed plasma concentration (C_max_) increased on days 1 and 15 with increasing pexidartinib doses, and time at C_max_ (T_max_) was consistent throughout all doses. Pexidartinib exposure and plasma levels of adiponectin and colony-stimulating factor 1 increased following multiple daily pexidartinib administrations. One patient (13%) with tenosynovial giant cell tumor showed objective tumor response. *Conclusions* This was the first study to evaluate pexidartinib in Asian patients with advanced solid tumors. Pexidartinib was safe and tolerable in this population at the recommended phase 2 dose previously determined for Western patients (funded by Daiichi Sankyo; clinicaltrials.gov number, NCT02734433).

## Introduction

Pexidartinib is a novel, orally administered small-molecule tyrosine kinase inhibitor (TKI) with strong selective activity against the colony-stimulating factor 1 receptor (CSF1R) [1]; the receptors KIT and FMS-like tyrosine kinase 3 internal tandem duplication mutation (FLT3-ITD) are also inhibited [2]. Based on these targets, pexidartinib may inhibit tumor growth directly by blocking the oncogenic drivers colony-stimulating factor 1 (CSF1), tyrosine protein kinase KIT (c-KIT), and FMS-like tyrosine kinase 3 (FLT3) [3–6], or indirectly by modulating the tumor microenvironment and affecting interactions between stromal and tumor cells [7–9]. Pexidartinib may also hinder tumor progression by blocking migration and angiogenesis of the tumor cell [10–12].

The utility of pexidartinib in treating patients with cancer was initially evaluated in a phase I study of Western patients with solid tumors [1]. In the dose-escalation part of the study (*n* = 41), grade 3 or higher treatment-related adverse events (AEs) occurred in 11/41 patients (27%), and those occurring in more than 1 patient included anemia, increase in aspartate aminotransferase (AST) level, and decrease in lymphocyte count (with each event occurring in 2 patients [5%]). A total of 8 dose-limiting toxicities (DLTs) occurred in 5 (12%) patients. The steady-state (day 15) median exposures, maximum observed plasma concentration (C_max_), and area under the plasma concentration-time curve from 0 to 24 h (AUC_0–24_) generally increased with increasing dose, and the median time to C_max_ (T_max_) values ranged from 1 to 2 h. In the extension part of study (*n* = 23), pexidartinib treatment resulted in a 52% overall response rate (ORR) and an 83% disease control rate by Response Evaluation Criteria in Solid Tumors version 1.1 (RECIST v1.1) in patients with recurrent or inoperable tenosynovial giant cell tumor (TGCT) [1].

This phase I, nonrandomized, open-label multiple-dose study is the first to evaluate the safety and pharmacokinetic profiles of pexidartinib in Asian patients with advanced solid tumors.

## Materials and methods

### Patients

Eligible patients were age 20 or older and had a histologically confirmed solid tumor that had relapsed from or was refractory to standard treatment, or for which standard treatment was not available. Female patients were required to have a negative serum pregnancy test within 14 days prior to treatment allocation, be surgically sterile, or be postmenopausal for ≥1 year; male and female patients of childbearing potential were required to use a highly effective contraception method throughout the study and for up to 90 days after completion. Additional inclusion criteria were Eastern Cooperative Oncology Group (ECOG) performance status of 0 or 1; life expectancy of ≥3 months; adequate hematologic, hepatic, and renal function; resolution (≤ grade 1 or baseline) of all toxicities from previous cancer therapy; and adequate treatment washout period before registration. Patients were excluded if they had previous use of pexidartinib or any biologic treatment targeting CSF1 or CSF1R, active primary central nervous system tumors or brain metastases, history of lung or heart disease (congestive heart failure, myocardial infarction, or unstable angina), or Fridericia-corrected QT interval (QTcF) ≥450 ms (men) or ≥ 470 ms (women). In addition, any patients with refractory nausea and vomiting, active or chronic infection with hepatitis C, active tuberculosis, or uncontrolled infection requiring intravenous injection of antibiotics, antivirals, or antifungals were excluded. All patients provided written informed consent.

### Study design and treatment

**Fig. 1:**
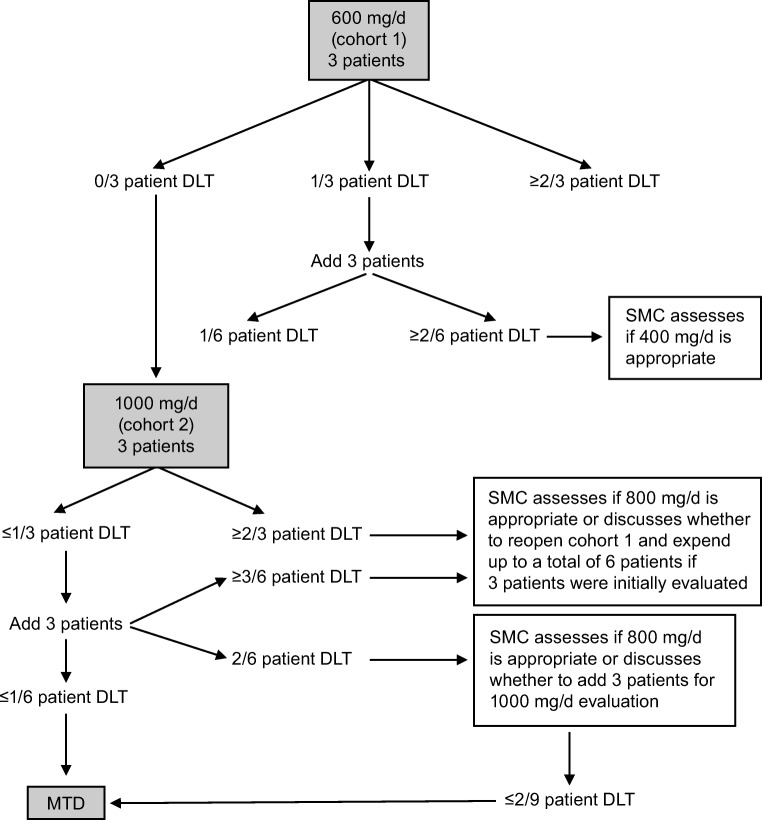
Schematic of overall study design and plan. DLT, dose-limiting toxicity; MTD, maximum tolerated dose; SMC, safety monitoring committee

This phase I, nonrandomized, open-label multiple-dose study was conducted at the National Taiwan University Hospital. The study had a dose-escalation 3 + 3 design (June 2016 to June 2017), comprising two dose levels (cohort 1 and cohort 2). Patients in cohort 1 received 600 mg/d (200 mg in the morning and 400 mg in the evening); patients in cohort 2 received 1000 mg/d (400 mg in the morning and 600 mg in the evening) for the first 2 weeks and 800 mg/d (400 mg twice per day, in the morning and evening) thereafter **(**Fig. 1**)**. The decrease in dose from 1000 to 800 mg/d after 2 weeks was based on a previous phase I study, in which many TGCT patients required dose modification [1]. Each treatment cycle was 28 days. Treatment continued until patient’s consent withdrawal, disease progression, or unacceptable toxicity. The maximum tolerated dose (MTD) was determined when no more than 1 of 6 patients experienced a DLT during cycle 1, which in turn determined the recommended phase 2 dose.

The Institutional Review Board at each participating center approved the study; ethics were in accordance with the Declaration of Helsinki and Good Clinical Practice guidelines of the International Conference on Harmonisation. The study is registered at ClinicalTrials.gov, number NCT02734433.

### Study objectives and endpoints

The primary objectives were to assess the safety and tolerability of pexidartinib and to determine the recommended phase 2 dose. The safety endpoints included treatment-emergent adverse events (TEAEs), serious adverse events (SAEs), DLTs, physical examination, vital sign measurements, standard clinical laboratory parameters, electrocardiographic parameters, and echocardiogram/multi-gated acquisition findings.

The secondary objectives were to evaluate the preliminary efficacy, pharmacokinetic profile, and pharmacodynamic effect on plasma levels of CSF1 and adiponectin of pexidartinib. The efficacy endpoints evaluated tumor response using RECIST v1.1, with the number and percentage of patients in each response category including complete response (CR), partial response (PR), stable disease (SD), progressive disease (PD), time to response, and percentage change in target lesions by magnetic resonance imaging (MRI). Time to response was defined as the time from the date of the first dose to the date at which criteria are first met for CR or PR. Duration of response for responders (CR or PR) was defined as the time interval between the date of the earliest qualifying response and the date of progressive disease or death from any cause, whichever occurs earlier. The pharmacokinetic endpoints evaluated AUC, C_max_, and T_max_, and the pharmacodynamic endpoints assessed the levels of CSF1 and adiponectin using plasma collected at various time points after pexidartinib administration. Selection of adiponectin as a marker of PD was based on clinically relevant 2- to 3-fold increases from baseline in patients treated with pexidartinib at dose levels of 900 mg/d and higher (PLX108–01 US phase I study, unpublished data, 2018).

### Study assessments and parameters

All TEAEs were graded according to the National Cancer Institute Common Terminology Criteria for Adverse Events (NCI-CTCAE) Version 4.03. The DLTs were defined as any drug-related TEAE that occurred during the first 28 days of treatment and met the criteria for hematologic toxicity (grade 4 anemia, neutropenia lasting >7 days, or platelet count decrease; grade ≥ 3 febrile neutropenia or platelet count decrease lasting >7 days or associated with bleeding), hepatic toxicity (grade 4 alanine aminotransferase [ALT] or AST increase, ALT or AST ≥3 × upper limit of normal [ULN] if accompanied by ≥2 × ULN in total bilirubin, or ALT or AST >5 × ULN lasting >14 days), and nonhematologic toxicity (grade ≥ 3 nonhematologic, nonhepatic major organ toxicity or inability to complete at least 75% of the pexidartinib prescribed dose in cycle 1 as a result of a drug-related TEAE).

Efficacy assessments (ORR, disease control rate, duration of response, duration of SD, time to response, and percentage of change in target lesions) were based on tumor assessments completed at screening and at every two cycles (±7 days) in the first four cycles after cycle 1 day 1 (C1D1) and thereafter at every three cycles (±7 days) if the patient remained on the study therapy.

A noncompartmental analysis was performed to determine pharmacokinetic parameters including C_max_, T_max_, and AUC from time 0 to 8 h (AUC_0-8h_) for pexidartinib and its metabolite (ZAAD-1006a). The pharmacodynamic analyses measured plasma levels of the CSF1 and adiponectin biomarkers at predose, cycle 1 day 15 (C1D15), and cycle 2 day 1 (C2D1).

### Statistical analysis

The data cutoff for the primary analysis (June 2017) occurred after all patients either discontinued the study or completed at least four cycles. After the primary analysis, the main study was closed, and the data were followed in the extension part of the study. For the assessment of tumor response, patients were classified, based on their best response, into the following categories: CR, PR, SD, progressive disease, or not evaluable. Exact binomial 95% confidence intervals (CIs; two-sided) were provided for each category response and the best ORR. The number of nonmissing observations, mean, standard deviation, median, minimum, and maximum as well as geometric means and geometric coefficient of variation were applied for C_max_ and AUC pharmacokinetic parameters. The T_max_ was summarized using median, maximum, and minimum values.

Assessments of change from baseline to posttreatment or the ratio of posttreatment to baseline included only patients with both baseline and posttreatment measurements. The last nonmissing value of a variable taken before the first dose of study drug was used as the baseline value, unless otherwise specified.

## Results

### Patients

**Fig. 2:**
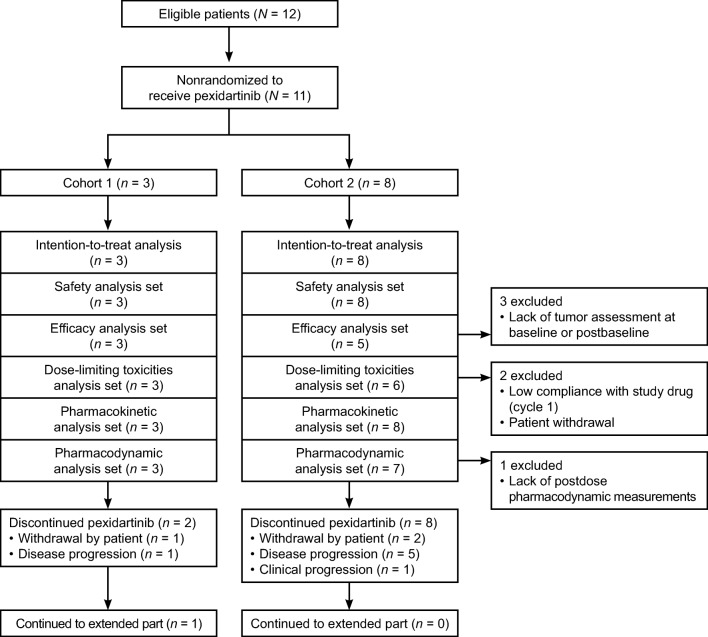
CONSORT diagram

Twelve patients were enrolled from one study center (National Taiwan University Hospital), 11 of whom (6 males and 5 females; median age 64, range 23–82) received pexidartinib; 1 patient was not treated due to a screen failure. Several tumor types were represented in the study, and baseline characteristics were summarized for cohort 1 (*n* = 3: renal cell carcinoma, TGCT, liver cancer) and cohort 2 (*n* = 8: sacral chordoma, malignant fibrous histiocytoma, bladder cancer, epithelioid trophoblastic tumor, submandibular gland, left adenoid cystic carcinoma, salivary gland cancer, large cell gallbladder neuroendocrine carcinoma, renal pelvic cancer) **(**see CONSORT diagram in Fig. 2**)**. The mean time since diagnosis of solid tumor type was 3.3 years, with a range of 1.7 to 6.6 years. Of the 11 patients, 9 had at least one prior tumor therapy (excluding surgery and radiation therapy) and concomitant analgesic use, while 6 patients had received radiation therapy **(**Table 1**)**. The TGCT patient from cohort 1 was a 23-year-old female who had prior surgeries (tendon sheath lesion excision and wrist surgery) and prior tumor therapy (oral metronomic cyclophosphamide and prednisolone), but no prior radiation therapy or prior concomitant analgesic use.

**Table 1:** Patient demographics and baseline disease characteristics

Characteristic	Cohort 1 *n* = 3	Cohort 2 *n* = 8	Total *N* = 11
Median age (range), year	68 (23–74)	63 (40–82)	64 (23–82)
Sex, *n* (%)			
Male	2 (67)	4 (50)	6 (55)
Female	1 (33)	4 (50)	5 (45)
Race, *n* (%)			
Asian	3 (100)	8 (100)	11 (100)
ECOG performance status at baseline, *n* (%)			
0	3 (100)	1 (13)	4 (36)
1	0	7 (88)	7 (64)
Type of solid tumor, *n* (%)			
Bladder cancer, urothelial carcinoma	0	1 (13)	1 (9)
Epithelioid trophoblastic tumor	0	1 (13)	1 (9)
Gallbladder neuroendocrine carcinoma, large cell type	0	1 (13)	1 (9)
Liver cancer	1 (33)	0	1 (9)
Malignant fibrous histiocytoma	0	1 (13)	1 (9)
Renal cell carcinoma	1 (33)	0	1 (9)
Renal pelvic cancer, right; urothelial carcinoma	0	1 (13)	1 (9)
Sacral chordoma	0	1 (13)	1 (9)
Salivary gland cancer, right submandibular pleiomorphic adenocarcinoma	0	1 (13)	1 (9)
Submandibular gland, left; adenoid cystic carcinoma	0	1 (13)	1 (9)
Tenosynovial giant cell tumor	1 (33)	0	1 (9)
Prior surgeries, *n* (%)			
1	0	1 (13)	1 (9)
2	1 (33)	3 (38)	4 (36)
≥3	2 (67)	4 (50)	6 (55)
Prior tumor therapy, *n* (%)			
Yes	2 (67)	7 (88)	9 (82)
No	1 (33)	1 (13)	2 (18)
Prior radiation therapy, *n* (%)			
Yes	2 (67)	4 (50)	6 (55)
No	1 (33)	4 (50)	5 (45)
Concomitant analgesic use, *n* (%)	2 (67)	7 (88)	9 (82)

*ECOG* Eastern Cooperative Oncology Group

### Safety

TEAEs of any grade occurred in all 11 patients (100%) who received pexidartinib at all dose levels, with 9 (82%) of the 11 experiencing a TEAE related to the drug, and 5 (45%) having at least one TEAE of grade 3 or 4. There was no dose-related trend with drug-related AEs of grade ≥ 3 **(**Table 2**)**. The most common TEAEs of any grade were AST increase in 5 patients (45%) and the following events in 4 patients (36%) each: ALT increase, fatigue, blood alkaline phosphatase (ALP) increase, and hair color change **(**Table 3**)**. The most common grade 3 or 4 AEs occurred in 18% of patients each (AST increase, blood ALP increase, gamma-glutamyl transferase increase, and anemia) **(**Table 3**)**.

**Table 2:** Summary of adverse events

	Cohort 1^a^ *n* = 3 *n* (%)	Cohort 2^b^ *n* = 8 *n* (%)	Total *N* = 11 *n* (%)
Number of patients with ≥1 TEAE	3 (100)	8 (100)	11 (100)
Number of patients with ≥1 drug-related TEAE	3 (100)	6 (75)	9 (82)
Number of patients with ≥1 serious TEAE	1 (33)	1 (13)	2 (18)
Number of patients with ≥1 drug-related serious TEAE	1 (33)	0	1 (9)
Number of patients with ≥1 TEAE grade 3/4	1 (33)	4 (50)	5 (45)
Number of patients with ≥1 drug-related TEAE grade 3/4	1 (33)	2 (25)	3 (27)
Number of patients who discontinued due to TEAE	0	0	0
Number of patients who died of TEAE	0	1 (13)	1 (9)

*TEAE* treatment-emergent adverse events

^a^Cohort 1: 600 mg/d (200 mg in the morning and 400 mg in the evening)

^b^Cohort 2: 1000 mg/d (400 mg in the morning and 600 mg in the evening) for the first 2 weeks. Thereafter, the dose was reduced to 800 mg/d (400 mg in the morning and 400 mg in the evening)

**Table 3:** Grade ≥ 3 adverse events in any patient or drug-related adverse events in >1 patient

Preferred term^a^	Any grade *N* = 11 *n* (%)	Grade ≥ 3 *N* = 11 *n* (%)	Drug-related any grade *N* = 11 *n* (%)
Aspartate aminotransferase increased	5 (45)	2 (18)	5 (45)
Alanine aminotransferase increased	4 (36)	1 (9)	4 (36)
Hair color changes	4 (36)	0	3 (27)
Fatigue	4 (36)	0	4 (36)
Blood alkaline phosphatase increased	4 (36)	2 (18)	4 (36)
Diarrhea	3 (27)	0	3 (27)
Gamma-glutamyl transferase increased	2 (18)	2 (18)	2 (18)
Anemia	2 (18)	2 (18)	0
Blood bilirubin increased	2 (18)	1 (9)	1 (9)
Back pain	2 (18)	1 (9)	0
Malignant neoplasm progression	1 (9)	1 (9)	0

^a^Classified according to the Common Terminology Criteria for Adverse Events criteria

Serious TEAEs occurred in 2 of 11 (18%) patients: 1 patient had increased ALT, AST, ALP, and blood bilirubin, and 1 patient experienced anemia as well as malignant neoplasm progression (grade 5), which resulted in death and was considered unrelated to the study drug. Other laboratory parameters that shifted from CTCAE grades 0–2 to grade 3 postbaseline included: lower lymphocytes (*n* = 3), lower hemoglobin and increased gamma-glutamyl transferase (*n* = 2), lower sodium (*n* = 1), and increased ALP and AST (*n* = 1). There were no changes in vital sign measurements. No DLTs were reported, with the MTD determined to be 1000 mg/d. The recommended phase 2 dose was 1000 mg/d for the first 2 weeks and 800 mg/d thereafter.

None of the treatment-related TEAEs in the current study resulted in discontinuation. Two patients (1 in each cohort) had the study drug interrupted due to TEAEs, and dose reduction was reported in 1 patient from each cohort. Four patients had treatment-emergent hepatotoxicity laboratory assessment abnormalities: AST/ALT >5 × ULN, AST/ALT >3 × ULN and concurrent total bilirubin >2 × ULN, AST/ALT >3 × ULN and AST/ALT ≤5 × ULN, and total bilirubin >2 × ULN **(**Table 4**)**. Of these abnormalities, only the increased bilirubin remained unrecovered or unresolved and was judged to be unrelated to study drug. None of the patients met Hy’s law criteria based on the safety monitoring committee review.

**Table 4:** Summary of TEAE hepatotoxicity laboratory assessment

Worst value during active treatment period	Cohort 1^a^ *n* = 3 *n* (%)	Cohort 2^b^ *n* = 8 *n* (%)	Total *N* = 11 *n* (%)
AST/ALT >3 × ULN and ≤ 5 × ULN	1 (33)	1 (13)	2 (18)
AST/ALT >5 × ULN	1 (33)	1 (13)	2 (18)
Total bilirubin >2 × ULN	1 (33)	1 (13)	2 (18)
AST/ALT >3 × ULN and concurrent total bilirubin >2 × ULN	1 (33)	0	1 (9)

*ALT* alanine aminotransferase, *AST* aspartate aminotransferase, *ULN* upper limit of normal

^a^Cohort 1: 600 mg/d (200 mg in the morning and 400 mg in the evening)

^b^Cohort 2: 1000 mg/d (400 mg in the morning and 600 mg in the evening) for the first 2 weeks. Thereafter, the dose was reduced to 800 mg/d (400 mg in the morning and 400 mg in the evening)

### Efficacy

**Fig. 3:**
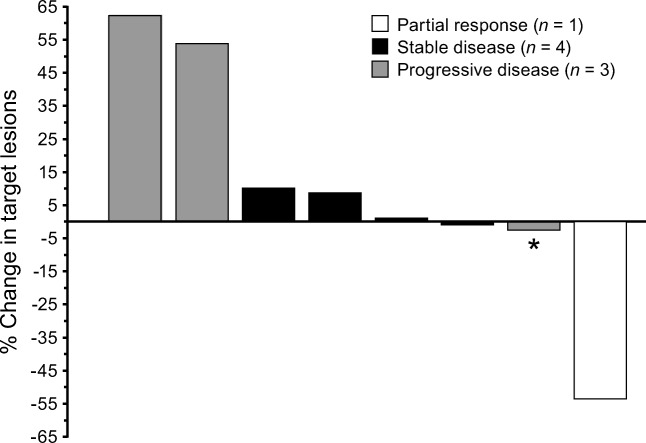
Percentage change in sum of longest diameters of target lesions from baseline. *The stable disease of this patient is only 53 days after first dosing date, so cannot be considered best overall response. Instead, this patient is classified as having progressive disease

**Fig. 4:**
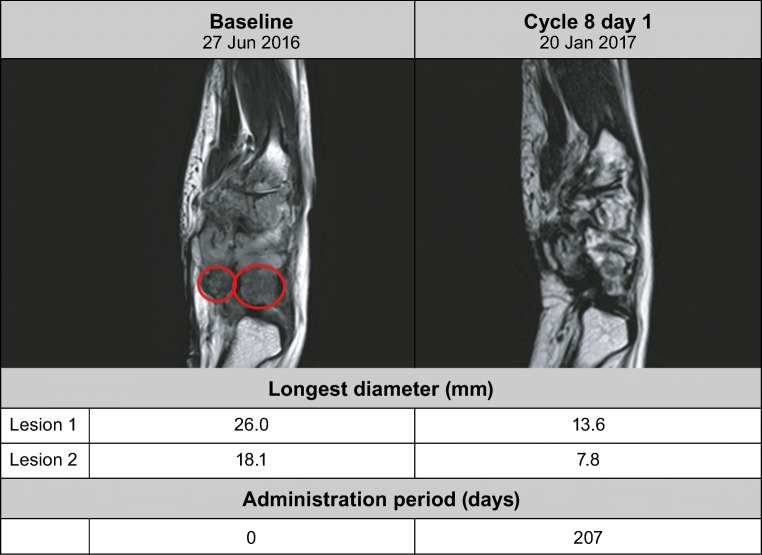
Longest diameter for right wrist synovial cavity by magnetic resonance imaging

The overall response rate (CR or PR) by RECIST was 13%, as the PR was found in 1 patient from cohort 1 with TGCT (Fig. 3). This patient continued into the extension part of the study, and nearly 7 months (207 days) into pexidartinib treatment had a large decrease in longest-diameter lesions (lesion 1, from 26.0 to 13.6 mm; lesion 2, from 18.1 to 7.8 mm) shown by MRI **(**Fig. 4**)**. The response was ongoing at 7.6 months in the TGCT patient who completed 13 cycles up to the cutoff date of the dose-escalation part of the study; the patient was still obtaining benefit from the study drug in the extension part of the study. The time to response for the TGCT patient from cohort 1 was 1.9 months. Overall, the disease control rate was 63% (5/8 patients; 67% [2/3] in cohort 1 and 60% [3/5] in cohort 2). There were 4 patients (50%) with SD with a mean duration of 3.9 months, and 3 (38%) patients with progressive disease.

The overall mean best percentage change from baseline in the sum of the longest diameters was 9.95% (range: −53.5-62.5%) **(**Fig. 3**).**

### Pharmacokinetics and pharmacodynamics

**Fig. 5:**
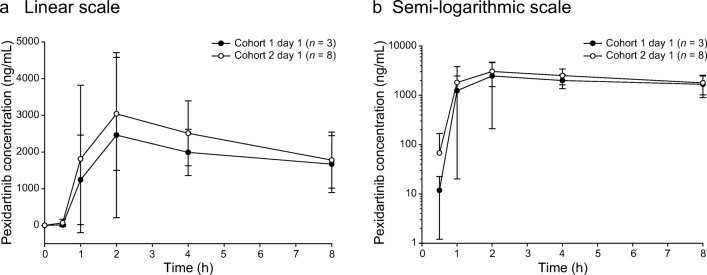
Mean (±STD) plasma concentrations of pexidartinib versus time—day 1. Cohorts 1 and 2: linear (**a**) and semi-logarithmic (**b**) scales

**Fig. 6:**
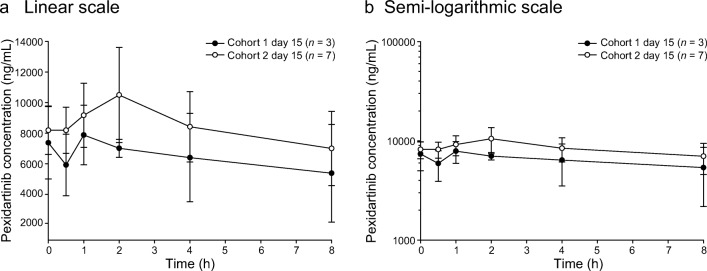
Mean (±STD) plasma concentrations of pexidartinib versus time—day 15. Cohorts 1 and 2: linear (**a**) and semi-logarithmic (**b**) scales

**Fig. 7:**
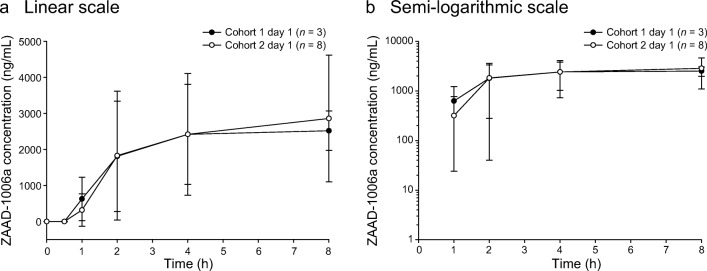
Mean (±STD) plasma concentrations of ZAAD-1006a versus time—day 1. Cohorts 1 and 2: linear (**a**) and semi-logarithmic (**b**) scales

**Fig. 8:**
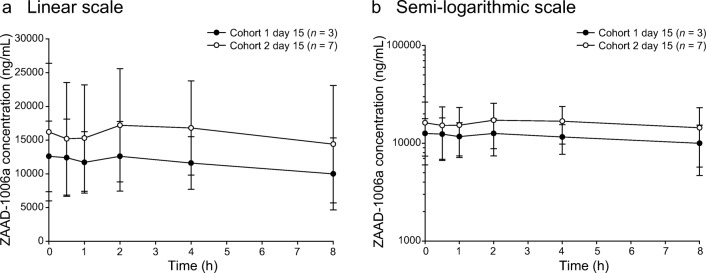
Mean (±STD) plasma concentrations of ZAAD-1006a versus time—day 15. Cohorts 1 and 2: linear (**a**) and semi-logarithmic (**b**) scales

For the pharmacokinetic analysis in both cohorts, the exposure parameters (AUC_0-8h_ and C_max_) increased on days 1 and 15 with increasing doses of pexidartinib, and T_max_ was consistent from 600 to 1000 mg/d, with the median ranging from 1.0 to 2.1 h. Pexidartinib plasma concentrations reached steady state within 8 days of twice-daily dose administration based on observation of trough concentrations. C_max_ and AUC_0-8h_ increased approximately 3- and 4-fold, respectively **(**Table 5**)**. Between cohorts, pexidartinib day 1 exposure (geometric mean AUC_0-8h_) increased by 1.24-fold and geometric mean C_max_ increased 1.13-fold, with an increase in daily dose from 600 to 1000 mg **(**Fig. 5 a and b). Day 15 exposure (geometric mean AUC_0-8h_) increased by 1.29-fold and geometric mean C_max_ increased 1.24-fold **(**Fig. 6 a and b). Geometric percent coefficient of variation (% CV) for the pexidartinib exposure parameters ranged from 16.4% to 39.6% across the two cohorts and study days. Following multiple oral administrations of pexidartinib, the total exposure (AUC_0-8h_) of the metabolite ZAAD-1006a was approximately 1.2-fold that of the parent drug at day 1 **(**Fig. 7 a and b) and day 15 **(**Fig. 8 a and b). Geometric % CV for the ZAAD-1006a exposure parameters ranged from 35.1% to 81.6% across the two cohorts and study days.

**Table 5:** Summary of pharmacokinetic parameters

Cohort	Day	C_max_ (ng/mL) geometric mean (% CV)	T_max_ (h) median (min, max)	AUC_0-8h_ (ng*h/mL) geometric mean (% CV)	R_Cmax_ geometric mean (% CV)	R_AUC_ geometric mean (% CV)
1	Cycle 1 day 1 (*n* = 3)	3050 (40)	2.1 (1.8–8.0)	12,800 (34)	–	–
	Cycle 1 day 15 (*n* = 3)	8420 (16)	1.0 (0.0–2.0)	48,900 (35)	2.8 (47)	3.8 (67)
2	Cycle 1 day 1 (*n* = 8)	3460 (39)	2.0 (0.8–4.0)	15,900 (30)	–	–
	Cycle 1 day 15 (*n* = 7)^a^	10,400 (31)	1.8 (0.0–2.1)	62,900 (26)	3.2 (57)	4.1 (52)

AUC_0-8h_, area under the plasma concentration-time curve from 0 to 8 h; C_max_, maximum plasma concentration; % CV, percent geometric coefficient of variation; R_AUC_, ratio of AUC_0-8h_ on cycle 1 day 15 to AUC_0-8h_ on cycle 1 day 1; R_Cmax_, ratio of C_max_ on cycle 1 day 15 to C_max_ on cycle 1 day 1; T_max_, time to reach C_max_

^a^One patient from cohort 2 was excluded from the pharmacokinetic analysis because of a lack of measurements

**Fig. 9:**
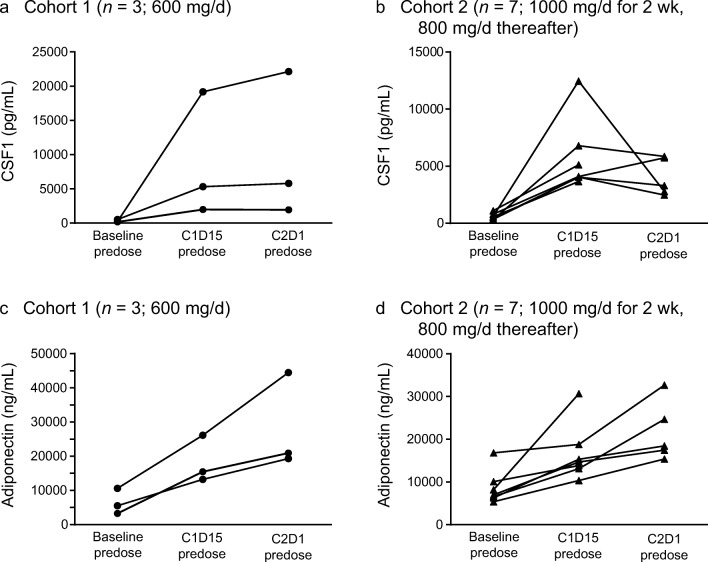
Individual CSF1 (**a**-**b**) and adiponectin (**c**-**d**) plasma levels by time. C, cycle; CSF1, colony-stimulating factor 1; D, day

In the pharmacodynamic analysis, following multiple-dose administration of pexidartinib, plasma concentrations of CSF1 increased with increasing dose. In cohort 1, the median CSF1 plasma concentration on C1D1 was 229.83 pg/mL. On C1D15, the median CSF1 increase from baseline was 984.12%, with no substantial further increase on C2D1 (median 971.51%) (Fig. 9a). In cohort 2, the median CSF1 plasma concentration on C1D1was 561.02 pg/mL. On C1D15, the median CSF1 increase from baseline was 918.58%, with no further apparent increase on C2D1 (median 627.26%) (Fig. 9b). In line with the CSF1 findings, plasma concentrations of adiponectin increased with dose following multiple-dose administration of pexidartinib. In cohort 1, the median adiponectin plasma concentration on C1D1 was 5525.51 ng/mL. On C1D15, the median adiponectin increase from baseline was 146.92%. Adiponectin plasma concentration continued to increase on C2D1 (median 320.18%) (Fig. 9c). In cohort 2, the median adiponectin plasma concentration on C1D1 was 6968.28 ng/mL. On C1D15, the median adiponectin increase from baseline was 99.34%. Adiponectin plasma concentration continued to increase on C2D1 (median 185.91%) (Fig. 9d). One patient from cohort 2 was excluded from the pharmacodynamic analysis set due to lack of postdose pharmacodynamic measurements.

## Discussion

This phase I, nonrandomized, open-label multiple-dose study was the first to evaluate pexidartinib in Asian patients with advanced solid tumors. Pexidartinib was safe and tolerable, with an MTD of 1000 mg/d and a recommended phase 2 dose of 1000 mg/d for the first 2 weeks and 800 mg/d thereafter, comparable to results from a previous study in Western patients [1]. Pexidartinib treatment resulted in an overall response of 13% with treatment continuing for over a year. Increases in AUC_0-8h_ and C_max_ were observed with increasing dose of pexidartinib treatment. Pexidartinib exposure increased after multiple doses, and plasma levels of biomarkers CSF1 and adiponectin increased after pexidartinib administration.

Pexidartinib was generally well tolerated; the most common TEAEs (≥20% overall incidence) were increased AST, ALT, and blood ALP; fatigue; hair color change; cough; and diarrhea. Five patients experienced grade ≥ 3 AEs, and there was no dose-related trend with the related drug grade ≥ 3 AEs, whereas in the Western population, 11 patients (27%) experienced drug-related grade ≥ 3 AEs [1]. Two patients experienced a total of 6 serious AEs during the dose-escalation phase, one of whom died of malignant neoplasm progression, which the investigator deemed to be unrelated to pexidartinib treatment. There were 4 patients who each experienced one treatment-emergent hepatotoxicity laboratory assessment abnormality. Of those, only 1 patient experienced increased bilirubin that remained unrecovered or unresolved; this was judged to be unrelated to the study drug and did not meet Hy’s law criteria as judged by the safety monitoring committee. No DLTs were reported in this study compared to the Western study, in which 5 patients experienced a total of 8 DLTs [1].

In this study, one patient with TGCT showed PR and continued pexidartinib treatment for more than 1 year. This rare disease is associated with pain, swelling, limitation of motion, and hemorrhagic joint effusions, which affect quality of life and are clinically important from the patient’s perspective [13]. In other studies, treatment with pexidartinib in TGCT patients resulted in an ORR of 52% (12/23) (phase I) [1] and 39% (24/61) (phase III) [14]. Response rates with other TKI agents have been inferior to the rate observed with pexidartinib. In a retrospective study of advanced TGCT patients treated with imatinib (*n* = 29), ORR was 19% (phase II) [15], whereas in a single-arm study of nilotinib (*n* = 56), ORR at week 12 was 0% (phase II) [16].

The current study showed that pexidartinib exposure increased following multiple daily doses of 600 mg (200 and 400 mg) or 1000 mg (400 and 600 mg), ranging from 3.84 to 4.14 for an accumulation ratio of AUC (R_AUC0-8h_), and from 2.76 to 3.24 for an accumulation ratio of C_max_ (R_Cmax_). In addition, plasma levels of adiponectin and CSF1 increased following multiple-dose administration of pexidartinib.

Strengths of the study included being the first safety and efficacy evaluation of pexidartinib in Asian patients and demonstrating tolerability and a pharmacokinetic profile comparable to those in previous findings in the Western population [1]. Our result also supports that pexidartinib may offer clinical benefit to TGCT patients. Further studies are needed to assess the role of pexidartinib in solid tumors to determine the optimized dosing schedule and combination regimens. Limitations of the study include the measurement of pharmacokinetic exposure of pexidartinib at 8 h, rather than 24 h used in the Western population [1]. In addition, only 1 patient achieved a response.

In conclusion, this study further establishes that pexidartinib was safe and tolerable in this population at the recommended phase 2 dose previously determined for Western patients, along with displaying a response in the lone TGCT patient.
